# The synergistic effect of diabetes mellitus and osteoporosis on the all-cause mortality: a cohort study of an American population

**DOI:** 10.3389/fendo.2023.1308574

**Published:** 2024-01-24

**Authors:** Weihua Li, Siyu Xie, Shengdong Zhong, Liting Lan

**Affiliations:** ^1^ Department of Orthopedics, Longyan First Affiliated Hospital of Fujian Medical University, Longyan, China; ^2^ Department of Anesthesiology, Longyan First Affiliated Hospital of Fujian Medical University, Longyan, China; ^3^ Department of Plastic Surgery, Longyan First Affiliated Hospital of Fujian Medical University, Longyan, China; ^4^ Clinical Research Center, The First Affiliated Hospital of Shantou University Medical College, Shantou, China

**Keywords:** diabetes, osteoporosis, interaction, all-cause death, NHANES

## Abstract

**Background:**

The increasing incidence of diabetes mellitus (DM) and osteoporosis have different effects on prognosis. The two often co-occur, so we aimed to investigate whether DM and osteoporosis have an effect on all-cause death and whether DM and osteoporosis have a synergistic effect.

**Methods:**

This study analyzed 18,658 subjects from five cycles of the National Health and Nutrition Examination Survey (NHANES). The primary endpoint was all-cause death. The subjects were divided into four groups based on the presence or absence of DM and osteoporosis. Survival curves and Cox regression analysis based on NHANES recommended weights were used to assess the risk of all-cause death between the diseased and non-diseased groups and to calculate additive interactions to assess whether there was a synergistic effect between diabetes and osteoporosis.

**Results:**

The group with DM and osteoporosis had the lowest survival rate. After full adjustment for confounders, patients with DM alone had a 30% higher risk of all-cause death compared with those without DM and osteoporosis (HR: 1.30, 95%CI: 1.09-1.55). Patients with osteoporosis alone had a 67% higher risk of all-cause death (HR: 1.67, 95%CI:1.16-2.43) and patients with combined DM and osteoporosis had a 127% higher risk of all-cause death (HR:2.27, 95%CI: 1.57-3.27). There was an additive interaction between DM and osteoporosis [RERI (95%CI): 1.03(0.55-1.50)] and excess mortality risk of 38% [AP (95% CI) 0.38(0.30-0.46)].

**Conclusions:**

There might be a synergistic effect of DM and osteoporosis on all-cause mortality, and patients with both conditions have a higher risk of death.

## Introduction

Diabetes mellitus (DM) presents a significant public health concern in both developed and developing nations. By 2021, 537 million people worldwide were affected (1). Osteoporosis is a long-term skeletal disorder distinguished by reduced bone density and degradation of the microscopic architecture of bone tissue. This results in heightened vulnerability of bones and a greater likelihood of fractures (2). It is estimated that more than 30 million people in Europe suffer from osteoporosis, and a similar number are affected in the United States (3, 4). DM is a multifaceted metabolic disorder which impacts various organs, leading to a range of complications including diabetic nephropathy, retinopathy, vasculopathy, and neuropathy. Likewise, osteoporosis places substantial burdens and constraints on individuals, manifesting as musculoskeletal system weakness and frailty. In relation to their occurrence in the general populace, both conditions are notable contributors to the development of disease complications. Extensive research in recent years has revealed a connection between DM and osteoporosis, whereby they may coexist as a result of metabolic disorders or iatrogenic effects, or even exhibit a cause-and-effect relationship during their progression (5–8).

Although osteoporosis has not traditionally been listed as a complication of DM, people with type 1 or type 2 DM are at increased risk of developing the disease. It has been widely accepted in previous research that DM has a direct impact on bone metabolism and strength, leading to gradual deterioration of bone microstructure and an elevated risk of developing osteoporosis (9). Furthermore, the increasing prevalence of both DM and osteoporosis raises concerns, especially regarding the potential drug-induced complications associated with corticosteroids, immunophilic proteins, and similar medications (9).

Many original papers, clinical statements, and guidelines have treated the management of patients with DM and osteoporosis as separate diseases, but it was unclear whether their co-existence increased their respective impact on patient prognosis, so we aimed to investigate whether there is an interaction for all-cause deaths when the two coexist.

## Method

### Study population

The source of the data is the National Health and Nutrition Examination Survey (NHANES) database, a nationally representative cross-sectional survey designed and conducted by the National Center for Health Statistics (NCHS). The survey uses a stratified, multistage probabilistic approach to sample the United States population and provides health and nutrition statistics on the noninstitutional civilian population of the United States. This is a large-scale probability survey of representatives of non-hospitalized civilian households in the United States, conducted annually and every two years in a cycle. This study used five cycles of the NHANSE dataset from 2005-2010, 2013-2014, and 2017-2018 for retrospective analysis, because these were the only cycles where bone density tests are performed. The NCHS Research Ethics Review Committee is mandated to investigate and verify that all participants provide informed consent. Detailed statistics see https://www.cdc.gov/nchs/nhanes/.

**Figure 1:**
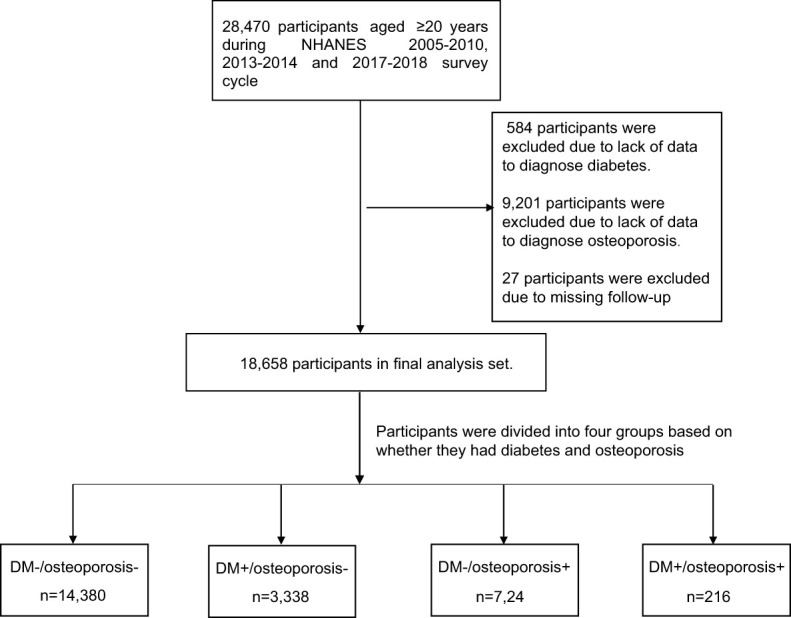
Flowchart of the study design.

In this cohort study, a population of 28,470 individuals aged 20 and above was enrolled. The identification of DM was established according to the diagnostic criteria recommended by the International Diabetes Association (IDM) and the prevailing clinical guidelines. DM can be determined by meeting any of the following criteria: (1) self-reported physician diagnosis of DM; (2) Glycosylated Hemoglobin, Type A1C (HbA1C,%) level is not less than 6.5%; (3) Fasting blood glucose level ≥7.0mmol/L; (4) Random blood glucose ≥11.1mmol/L; (5) Oral glucose tolerance test (OGTT) 2 hours blood glucose ≥11.1mmol/L; (6) Receiving oral hypoglycemic drugs or insulin therapy. Bone mass density (BMD) (g/cm 2) of the subjects was examined using DXA. Osteoporosis was diagnosed using World Health Organization criteria, defined as bone mineral density at the neck of the femur equal to or less than 2.5 standard deviations of the average for young people of the same sex. We excluded patients who lacked a DM diagnosis (n= 584) and an osteoporosis diagnosis (n= 9,201). In addition, 27 participants with failed follow-up were excluded. In the end, the retrospective study included a total of 18,658 participants (**Figure 1**).

### Covariates

In NHANES, data were collected through a standard participant questionnaire conducted during the in-home interview process and a medical assessment of each participant. The covariates considered in this study included age, gender, race, education, smoking status, alcohol consumption, obesity, exercise metabolic equivalent, HbA1C, calcium, phosphorus, hyperlipidemia, self-reported hypertension, self-reported chronic kidney disease (CKD), and self-reported cardiovascular disease (CVD). For smoking status, participants were considered smokers if they had smoked 100 or more cigarettes in the past. Participants who had smoked fewer than 100 cigarettes in the past were considered non-smokers. For drinking status, participants were classified as non-drinkers and drinkers. The definition of obesity was assessed by body mass index (BMI≥30), height was measured using an electronic motion-measuring instrument with an accuracy of millimeters, and researchers used a digital scale to measure weight and convert pounds to kilograms when the measurement was completed. Hyperlipidemia is defined by the National Cholesterol Education Program (NCEP) as triglycerides ≥ 150 mg/dL, total cholesterol ≥ 200 mg/dL, low-density lipoprotein ≥ 130 mg/dL, or HDL ≤ 40 mg/dL for women, and ≤ 50 mg/dL for women. In addition, participants who reported using cholesterol-lowering drugs were also defined as having hyperlipidemia. The description of each variable see https://www.cdc.gov/Nchs/Nhanes/continuousnhanes/.

### Statistical analysis

We used the weights recommended by the NHANES to calculate the group-specific weights. Continuous variables were expressed as mean (standard error) and categorical variables were expressed as counts (percentages). Baseline characteristics were analyzed by ANOVA and chi-square test for continuous and categorical variables respectively.

Kaplan-Meier survival analysis curves and Cox regression analyses were used to assess hazard ratios (HR) and 95% confidence interval (CI) for participants with DM or osteoporosis alone and for participants with both DM and osteoporosis relative to the risk of all-cause death without DM and osteoporosis. The first model describes unadjusted associations. The second model is to adjust for age, gender and race. The third model controlled for age, gender, race, education, smoke, alcohol consumption, obese, exercise metabolic equivalent, phosphorus, HbA1C, hyperlipidemia, CKD, hypertension and CVD. We also performed a stratified analysis based on subgroups (age, gender, hyperlipidemia, hypertension, CKD, and CVD) to assess differences between subgroups. In the subgroup of women, menopause was added as a factor to the regression model. Additive interactions were studied by calculating the relative excess risk of the interaction (RERI), the attributive proportion of the interaction (AP), and the concomitant 95% CI to determine whether there was a synergistic effect between diabetes and osteoporosis. To try to avoid reverse causality, we also removed patients who died within one year of follow-up for sensitivity analysis.

All data were obtained using R Studio (version 4.2.1), two-sided P values <0.05 indicated significance in all analyses.

## Result

### Baseline characteristics

Baseline characteristics differed between the exposed groups (**Table 1**). There was no significant difference in serum calcium level among all groups. Compared with the other groups, people with DM alone group smoked and drank more, were more likely to be co-obese, and were more likely to have hyperlipidemia. people with osteoporosis alone group with osteoporosis had the highest blood phosphorus levels. The group with osteoporosis alone was older, had a higher proportion of women, had a higher proportion of Hispanic whites, and had the highest blood phosphorus levels. Compared with other groups, the group with DM and osteoporosis had the lowest education level, the highest exercise metabolic equivalent, is more likely to have hypertension, CKD, CVD and other comorbidities, and the all-cause mortality is the highest (DM-/osteoporosis-: 7.0% *vs*. DM+/osteoporosis-: 17.8% *vs*. DM-/osteoporosis+: 23.7% *vs*. DM+/osteoporosis+: 40.8%).

**Table 1:** Baseline study population characteristics (weighted).

Variable	DM-/osteoporosis-N= 14,380	DM+/osteoporosis-N= 3,338	DM-/osteoporosis+N= 724	DM+/osteoporosis+N= 216		P-value	
Age, mean (SE).	48.0(0.3)	60.4(0.3)	63.7(0.7)	67.2(1.0)		< 0.001	
Female, n (%)	6,968(50.0)	1,441(43.5)	520(73.3)	154(70.8)		< 0.001	
Race, n (%)						< 0.001	
Mexican American	2,325(7.4)	653(9.4)	98(6.1)	43(9.8)			
Non-Hispanic Black	2,802(10.0)	790(13.5)	48(3.2)	19(5.6)			
Non-Hispanic White	6,980(71.7)	1,245(63.5)	433(79.1)	93(63.9)			
Other Hispanic	1,264(4.6)	349(5.7)	52(3.7)	24(6.8)			
Other Race -	1,009(6.3)	301(7.8)	93(7.9)	37(13.8)			
Education, n (%)						< 0.001	
Grade 12 and below	3,461(15.5)	1,154(22.4)	223(22.0)	96(32.9)			
High School Grad	3,399(23.9)	779(26.0)	185(26.6)	58(30.7)			
College or AA degree	7,506(60.6)	1,397(51.6)	316(51.4)	61(36.4)			
HbA1C, (%)	5.4(0.0)	7.0(0.0)	5.5(0.0)	6.9(0.1)		< 0.001	
Smoke, n (%)	6,753(46.4)	1,694(50.3)	312(47.5)	86(40.1)		0.030
Alcohol Consumption, n (%)	9,677(77.7)	1,705(63.7)	335(60.4)	71(43.0)		< 0.001	
Obese, n (%)	4,444(30.3)	1,686(56.1)	115(17.2)	63(35.8)		< 0.001	
Menopause, n (%)	3,786(26.6)	1,253(37.1)	495(70.0)	153(70.2)		< 0.001	
Exercise metabolic equivalent, MET/week	3,825.7(105.8)	3,260.4(182.7)	2,600.4(201.1)	3,087.3(838.9)		< 0.001	
Calcium, mg/dl	9.4(0.0)	9.4(0.0)	9.4(0.0)	9.4(0.0)		0.590	
Phosphorus, mg/dl	3.8(0.0)	3.7(0.0)	3.8(0.0)	3.8(0.0)		< 0.001	
Hyperlipidemia, n (%)	9,900(69.5)	2,862(88.2)	540(76.2)	172(82.5)		< 0.001	
Hypertension, n (%)	5,522(34.5)	2,369(69.3)	396(52.7)	160(76.3)		< 0.001	
CKD, n (%)	1,903(11.1)	1,247(35.2)	207(27.7)	103(53.0)		< 0.001	
CVD, n (%)	1,225(6.8)	817(23.9)	123(14.2)	65(34.2)		< 0.001	
All-cause death	1,399(7.0)	715(17.8)	200(23.7)	88(40.8)		< 0.001	

Values are, n (%) or mean (SE).

Glycosylated Hemoglobin, Type A1C, HbA1c; CKD, Chronic kidney disease; CVD, cardiovascular disease.

### Associations with all-cause mortality

**Figure 2:**
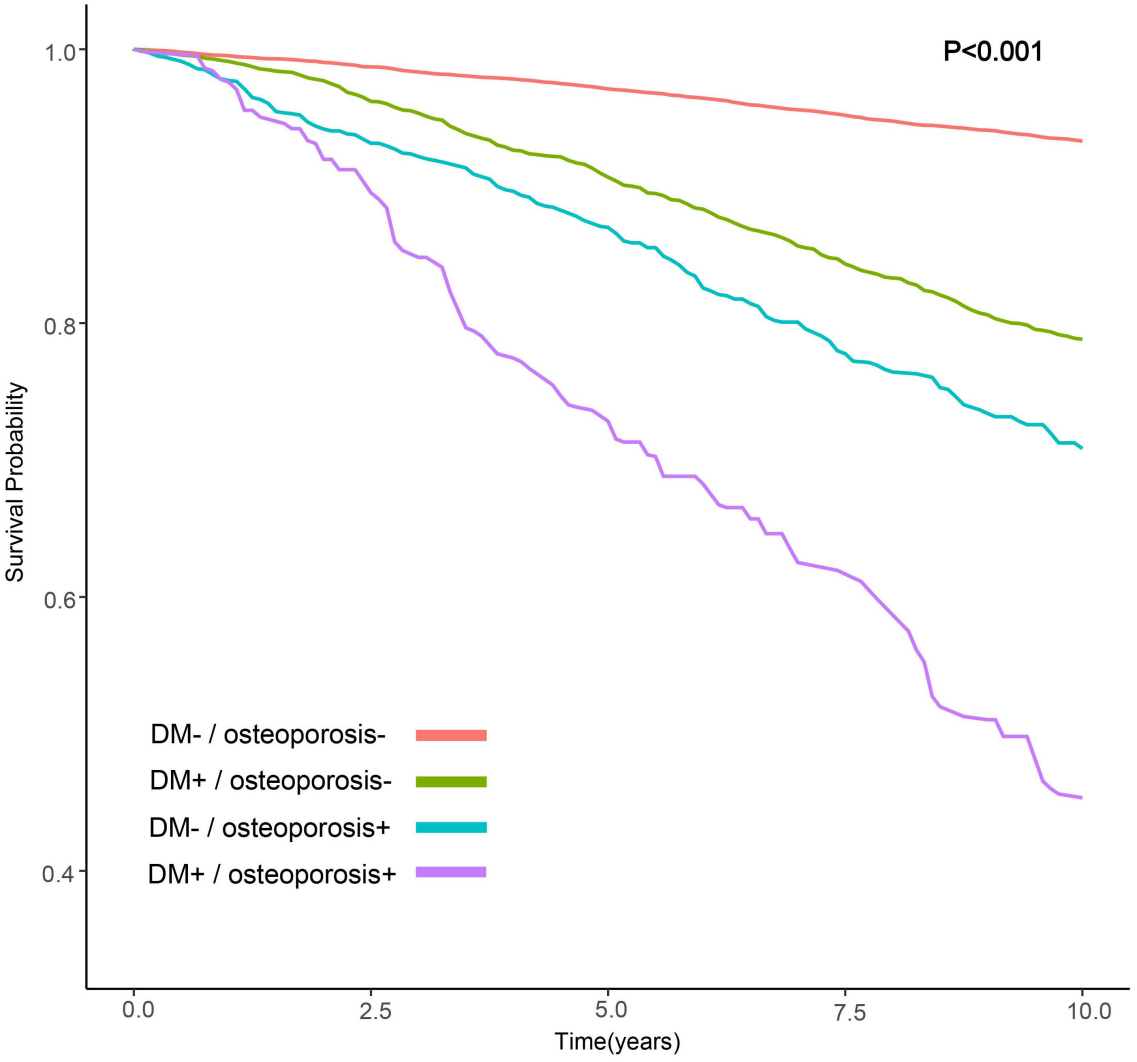
Kaplan-Meier survival estimates for all-cause mortality (weighted).

We observed that the group with DM and osteoporosis had the lowest survival rate. Kaplan-Meier survival analysis curves showed that the groups without DM and osteoporosis had the highest survival rate, followed by the group with DM alone, followed by the group with osteoporosis alone, and the group with DM and osteoporosis had the lowest survival rate (P-log rank < 0.001, **Figure 2**). In all models, patients with DM alone, patients with osteoporosis alone, patients with DM and osteoporosis were associated with a greater risk of all-cause mortality than patients without DM and osteoporosis (**Table 2**). After adjusting for various factors, the association weakened somewhat but was still statistically significant. In the fully adjusted model, the risk of all-cause death was 30% higher in people with DM alone (HR: 1.30; 95% CI 1.09-1.55), 67% higher in people with osteoporosis alone (HR:1.67; 95% CI: 1.16-2.43) and 127% higher in patients with DM and osteoporosis (HR: 2.27, 95% CI: 1.57-3.27) compared with patients without DM and osteoporosis. Associations in all models followed the same pattern, with the greatest effect for patients with DM and osteoporosis. For patients with DM alone, the effect was minimum.

**Table 2:** The association between diabetes and osteoporosis and all-cause death (weighted).

Group	Model 1		Model 2		Model 3	
	OR (95%CI)	*P-value*	OR (95%CI)	*P-value*	OR (95%CI)	*P-value*
DM-/osteoporosis-	Ref		Ref		Ref	
DM+/osteoporosis-	3.38(3.02-3.79)	<0.001	1.54(1.39-1.72)	<0.001	1.30(1.09,1.55)	<0.001
DM-/osteoporosis+	4.75(3.74-6.04)	<0.001	1.84(1.45-2.34)	<0.001	1.67(1.16,2.43)	<0.001
DM+/osteoporosis+	10.31(7.43-14.29)	<0.001	3.58(2.64-4.86)	<0.001	2.27(1.57,3.27)	<0.001
**Additive interaction**						
RERI (95%CI) for DM and osteoporosis: 1.03(0.55-1.50)						
AP (95%CI) for DM and osteoporosis: 0.38(0.30-0.46)						

Model 1: Not adjusted.

Model 2: Adjusted by age, gender, race/ethnicity.

Model 3: Adjusted by age, gender, race/ethnicity, education, smoke, drink, obese, Exercise metabolic equivalent, HbA1C, Phosphorus, Hyperlipidemia, CKD, Hypertension, CVD.

### Additive interaction

The RERI of interaction between DM and osteoporosis was 1.03[95%CI: 0.55-1.50], indicating an interaction on an additive scale, with the risk of all-cause death in the DM and osteoporosis comorbidities exceeding the combined risk of DM and osteoporosis alone. The attribution proportion due to AP was 0.38 [95% CI: 0.30-0.46] (**Table 2**).

### Subgroup analysis

**Figure 3:**
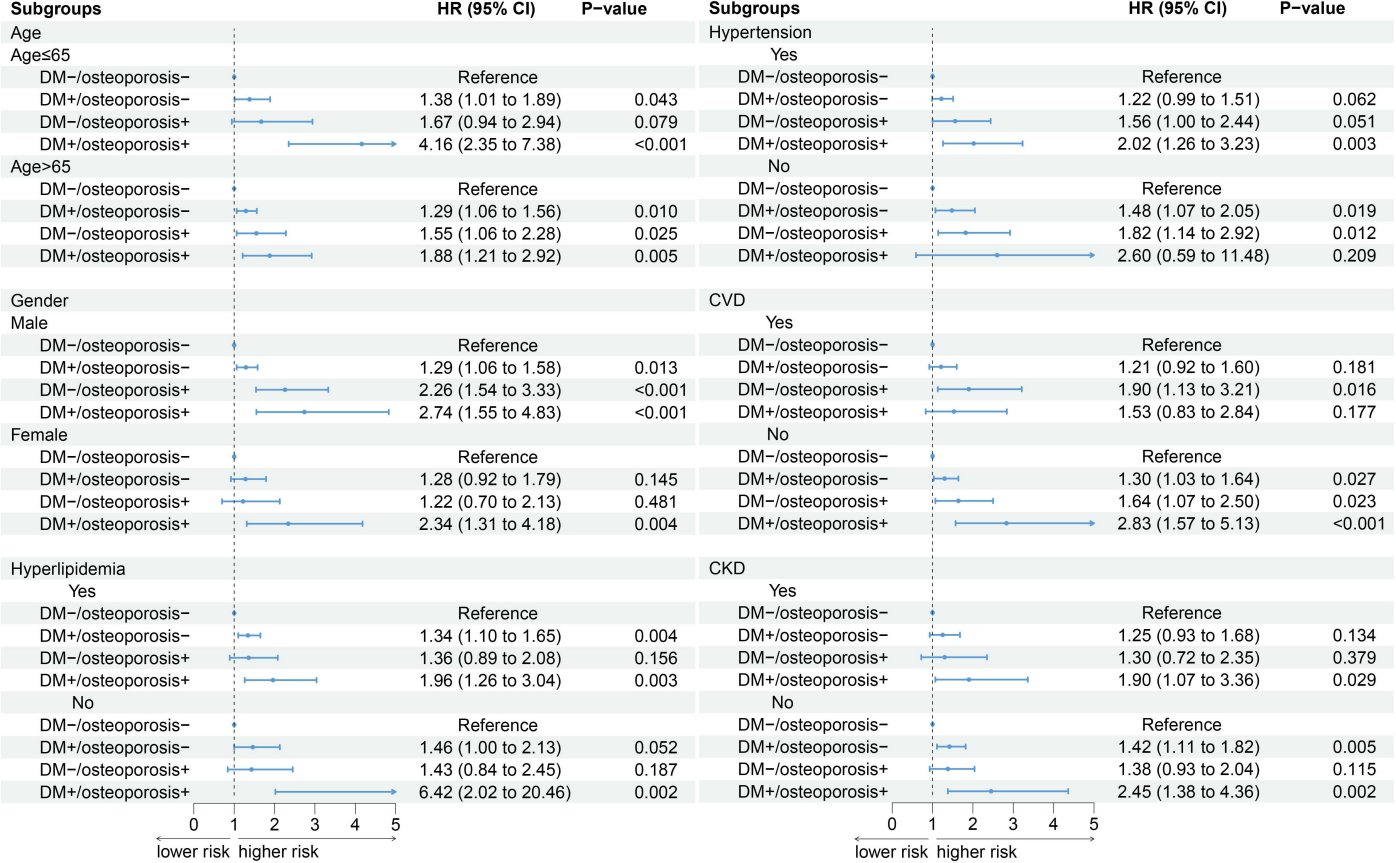
Subgroup analysis. An additional adjustment for “menopause” was made in the female subgroup, and the remaining subgroups were adjusted according to model 3.

When participants were stratified by age, gender, hyperlipidemia, and CKD, the relative risk of death was highest in those with combined diabetes and osteoporosis compared with those without diabetes and osteoporosis (all p <0.05). When stratified by hypertension and cardiovascular disease, there was no significant difference in death between groups without hypertension and those with CVD (**Figure 3**).

### Sensitivity analyses

Similar results were found in the sensitivity analysis. Kaplan-Meier survival analysis curve showed that the survival rate was highest in the group without DM and osteoporosis, followed by the DM alone group, followed by the osteoporosis alone group, and the survival rate was lowest in the group combined with DM and osteoporosis (P-log rank < 0.001, **Figure S1**). Compared with patients without DM and osteoporosis, those with DM alone had a 35% higher risk of all-cause death (HR: 1.35; 95% CI: 1.19-1.54). Patients with osteoporosis alone had a 66% higher risk of all-cause death (HR: 1.66; 95% CI: 1.25-2.21). Patients with combined DM and osteoporosis were 182% higher (HR: 2.82; 95% CI: 2.05-3.88). There was an additive interaction between DM and osteoporosis (RERI (95%CI): 0.64(0.36-0.92)), and excess mortality risk of 0.27 [AP (95%CI): 0.27(0.20-0.35)] (**Table S1**).

## Discussion

In this large cross-sectional study in the United States, our results confirmed the higher risk of death associated with DM and osteoporosis and also found that the synergistic effect of DM and osteoporosis on all-cause death exceeded expectations for their respective effects, a pattern that persisted even after adjusting for various confounding factors. Results remained stable after stratification for age, gender, hyperlipidemia, hypertension, CVD, and CKD.

DM and osteoporosis are global health concerns due to their high incidence in the general population, especially in the elderly. Although osteoporosis is not generally considered a complication of DM, emerging evidence suggests that osteoporosis is increasingly prevalent in people with type 2 diabetes mellitus (T2D). Both T2D and osteoporosis are associated with significant morbidity, higher mortality, and higher social costs due to their chronic consequences (10). Epidemiological studies have shown that T2D is associated with an increased risk of fracture, highlighting the need to consider bone fragility as a chronic complication of T2D (11, 12). Instead, T2D should be considered a factor in endocrine-related osteoporosis (13). Osteoporosis-related fractures, especially those of the spine and hip, often result in chronic pain, disability, and reduced quality of life. Such fractures require hospitalization, increase the risk of death by 20%, and lead to chronic disability in up to 50% of cases (14). The co-existence of DM complications and the risk of falls further exacerbates fractures (15). Therefore, more attention must be paid to the coexistence of these two conditions. However, limited studies have explored the effect on all-cause mortality when DM and osteoporosis co-exist. Our research investigation found a higher risk of all-cause death when DM and osteoporosis co-existed compared to when they occurred alone. In addition, the additive interaction model also showed a synergistic effect of DM and osteoporosis on all-cause mortality

There may be some common pathways leading to poor prognosis between the two diseases. Age and obesity may be important factors. A large-scale study conducted across multiple centers revealed a noteworthy increase in the prevalence of osteoporosis among men and women aged 55 and above, as demonstrated by measurements of lumbar spine, femoral neck, and total femoral BMD (16). Considering that senescent cells accumulate universally in various tissues with age, numerous cell types in the bone microenvironment are also prone to cellular aging in different scenarios. Aging osteoblasts and osteoclasts release crucial factors that regulate osteoclast function, suggesting that aging osteoblasts directly contribute to the development of age-related osteoporosis (17). T2D can induce various manifestations of accelerated aging in humans, and senescent cells have been observed to accumulate at an early stage of life in multiple tissues, potentially including fat, liver, pancreas, brain, and bone (18). Furthermore, the rise of obesity is a prevailing characteristic observed in T2D, contributing to intricate impacts on bones that can be either advantageous or detrimental. For instance, although the elevated body weight and abundance of lean soft tissue mass correlated with obesity and T2D can exert a favorable mechanical loading influence on weight-bearing bones, the concomitant escalation in circulating adipokines and pro-inflammatory cytokines, particularly those discharged from visceral adipose tissue reservoirs, has the potential to intensify mechanical loading on skeletal sites, ultimately promoting bone resorption (19, 20). Obesity and T2D can also contribute to the buildup of fat in the bone marrow, potentially leading to detrimental effects on the surrounding bone microenvironment. These effects may include impairments in bone formation. However, further investigation is necessary to gain a more comprehensive understanding of the precise impact of bone marrow fat on bone formation. In addition, a study noted that the effect of osteoporosis on carbohydrate metabolism is significant, especially in individuals diagnosed with DM (21). Because of the interaction between DM and osteoporosis, we suspect that the higher death risk of patients with both diseases is not the result of the superposition of the death risks of the two diseases. As shown by the results of our additive interaction model, there may be a synergistic effect between the two diseases.

Our findings indicate a potential interplay between DM and osteoporosis, leading to an elevated likelihood of overall mortality. As a result, it is crucial to adopt proactive measures in clinical settings aimed at regulating blood glucose levels and averting the onset of osteoporosis. Notably, risk factors such as smoking habits and frequency of physical activity should be closely monitored among individuals simultaneously affected by DM and osteoporosis. Scientific research has shown that metformin, among the hypoglycemic drugs available, offers advantages in preserving bone mineral density in individuals with DM. Conversely, it is not advisable for diabetic patients with bone mineral disorders to take sulfonylureas (due to the potential for hypoglycemia) or thiazolidinediones (due to their mechanism of action). The impact of newer antidiabetic medications like sodium-glucose cotransporter-2 (SGLT2) inhibitors and dipeptidyl peptidase 4 (DPP4) inhibitors on bone health and DM remains uncertain, as there is insufficient data available (21).

There are some limitations to our study. Our study is a retrospective analysis of NHANES, and confounding factors may exist, so we conducted cox regression. Next, we plan to conduct further cohort studies or randomized controlled trials. Moreover, cross-sectional studies fail to verify cause-and-effect relationships. Third, self-reported questionnaires may lead to recall bias or reporting bias.

## Conclusion

There is a significant interaction between DM and osteoporosis, leading to an increased risk of subsequent all-cause mortality events. Our findings highlight the management and prevention of outcomes in patients with DM and osteoporosis to reduce the risk of all-cause mortality.

## Availability of data and materials

All data are available as publicly accessible datasets through NHANES. It is open and publicly accessible through the following link; https://wwwn.cdc.gov/nchs/nhanes/.

## Ethics approval and consent to participate

Informed consent has been obtained from every participant and therefore there was no need for any ethical consent in this study. The NCHS ethics review board has approved the NHANES protocol. All procedures were performed in accordance with the relevant guidelines and regulations.

## Data availability statement

The raw data supporting the conclusions of this article will be made available by the authors, without undue reservation.

## Ethics statement

The requirement of ethical approval was waived by The NCHS ethics review board has approved the NHANES protocol. for the studies on humans because Informed consent has been obtained from every participant and therefore there was no need for any ethical consent in this study. The NCHS ethics review board has approved the NHANES protocol. All procedures were performed in accordance with the relevant guidelines and regulations. The studies were conducted in accordance with the local legislation and institutional requirements. Written informed consent for participation was not required from the participants or the participants’ legal guardians/next of kin in accordance with the national legislation and institutional requirements. The human samples used in this study were acquired from gifted from another research group.

## Author contributions

WL: Writing – original draft. SX: Writing – review & editing. SZ: Writing – review & editing. LL: Writing – review & editing.

## Supplementary material

Supplementary Figure 1 Kaplan-Meier survival estimates for all-cause mortality (sensitivity analysis, weighted).

## References

[1] Sun H Saeedi P Karuranga S Pinkepank M Ogurtsova K Duncan BB. IDF Diabetes Atlas: Global, regional and country-level diabetes prevalence estimates for 2021 and projections for 2045. Diabetes Res Clin Pract (2022) 183:109119. doi: 10.1016/j.diabres.2021.109119 34879977 PMC11057359

[2] Armas LAG Recker RR. Pathophysiology of osteoporosis: new mechanistic insights. Endocrinol Metab Clin North Am (2012) 41(3):475–86. doi: 10.1016/j.ecl.2012.04.006 22877425

[3] Wright NC Looker AC Saag KG Curtis JR Delzell ES Randall S. The recent prevalence of osteoporosis and low bone mass in the United States based on bone mineral density at the femoral neck or lumbar spine. J Bone Miner Res (2014) 29(11):2520–6. doi: 10.1002/jbmr.2269 PMC4757905 24771492

[4] Johnell O Kanis JA. An estimate of the worldwide prevalence and disability associated with osteoporotic fractures. Osteoporos Int (2006) 17(12):1726–33. doi: 10.1007/s00198-006-0172-4 16983459

[5] Wong SK Chin K-Y Suhaimi FH Ahmad F Ima-Nirwana S. The relationship between metabolic syndrome and osteoporosis: A review. Nutrients (2016) 8(6):347. doi: 10.3390/nu8060347 27338453 PMC4924188

[6] Adil C Aydın T Taşpınar Ö Kızıltan H Eriş AH Hocaoglu IT. Bone mineral density evaluation of patients with type 2 diabetes mellitus. J Phys Ther Sci (2015) 27(1):179–82. doi: 10.1589/jpts.27.179 PMC4305556 25642068

[7] Whittier X Saag KG. Glucocorticoid-induced osteoporosis. Rheum Dis Clin North Am (2016) 42(1):177–89. doi: 10.1016/j.rdc.2015.08.005 26611558

[8] Katsuyama T Sada K-E Namba S Watanabe H Katsuyama E Yamanari T. Risk factors for the development of glucocorticoid-induced diabetes mellitus. Diabetes Res Clin Pract (2015) 108(2):273–9. doi: 10.1016/j.diabres.2015.02.010 25765669

[9] Nguyen K-D Bagheri B Bagheri H. Drug-induced bone loss: a major safety concern in Europe. Expert Opin Drug Saf (2018) 17(10):1005–14. doi: 10.1080/14740338.2018.1524868 30222369

[10] Valderrábano RJ Linares MI. Diabetes mellitus and bone health: epidemiology, etiology and implications for fracture risk stratification. Clin Diabetes Endocrinol (2018) 4:9. doi: 10.1186/s40842-018-0060-9 29721333 PMC5918531

[11] Fan Y Wei F Lang Y Liu Y. Diabetes mellitus and risk of hip fractures: a meta-analysis. Osteoporos Int (2016) 27(1):219–28. doi: 10.1007/s00198-015-3279-7 26264604

[12] Russo GT Giandalia A Romeo EL Nunziata M Muscianisi M Ruffo MC. Fracture risk in type 2 diabetes: current perspectives and gender differences. Int J Endocrinol (2016) 2016:1615735. doi: 10.1155/2016/1615735 28044077 PMC5164892

[13] Eller-Vainicher C Falchetti A Gennari L Cairoli E Bertoldo F Vescini F. DIAGNOSIS OF ENDOCRINE DISEASE: Evaluation of bone fragility in endocrine disorders. Eur J Endocrinol (2019) EJE-18-0991.R1. doi: 10.1530/EJE-18-0991 31042675

[14] Liu J Curtis EM Cooper C Harvey NC. State of the art in osteoporosis risk assessment and treatment. J Endocrinol Invest (2019) 42(10):1149–64. doi: 10.1007/s40618-019-01041-6 PMC6751157 30980341

[15] Dede AD Tournis S Dontas I Trovas G. Type 2 diabetes mellitus and fracture risk. Metabolism (2014) 63(12):1480–90. doi: 10.1016/j.metabol.2014.09.002 25284729

[16] Zeng Q Li N Wang Q Feng J Sun D Zhang Q. et al: the prevalence of osteoporosis in China, a nationwide, multicenter DXA survey. J Bone Miner Res (2019) 34(10):1789–97. doi: 10.1002/jbmr.3757 31067339

[17] Farr JN Xu M Weivoda MM Monroe DG Fraser DG Onken JL. et al: Targeting cellular senescence prevents age-related bone loss in mice. Nat Med (2017) 23(9):1072–9. doi: 10.1038/nm.4385 PMC5657592 28825716

[18] Farr JN. Skeletal senescence with aging and type 2 diabetes. Endocrinol Metab (Seoul) (2023) 38(3):295–301. doi: 10.3803/EnM.2023.1727 37312256 PMC10323162

[19] Napoli N Chandran M Pierroz DD Abrahamsen B Schwartz AV Ferrari SL. Mechanisms of diabetes mellitus-induced bone fragility. Nat Rev Endocrinol (2017) 13(4):208–19. doi: 10.1038/nrendo.2016.153 27658727

[20] Khosla S Samakkarnthai P Monroe DG Farr JN. Update on the pathogenesis and treatment of skeletal fragility in type 2 diabetes mellitus. Nat Rev Endocrinol (2021) 17(11):685–97. doi: 10.1038/s41574-021-00555-5 PMC8605611 34518671

[21] Zawada A Ratajczak AE Rychter AM Szymczak-Tomczak A Dobrowolska A. Krela-kaźmierczak I: treatment of diabetes and osteoporosis-A reciprocal risk? Biomedicines (2022) 10(9):2191. doi: 10.3390/biomedicines10092191 36140292 PMC9495959

