# Supplementary figures and images for: The synergistic effect of diabetes mellitus and osteoporosis on the all-cause mortality: a cohort study of an American population
Source: Front Endocrinol (Lausanne). 2024 Jan 24;14:1308574. doi: 10.3389/fendo.2023.1308574 (PMC10849060; doi:10.3389/fendo.2023.1308574)

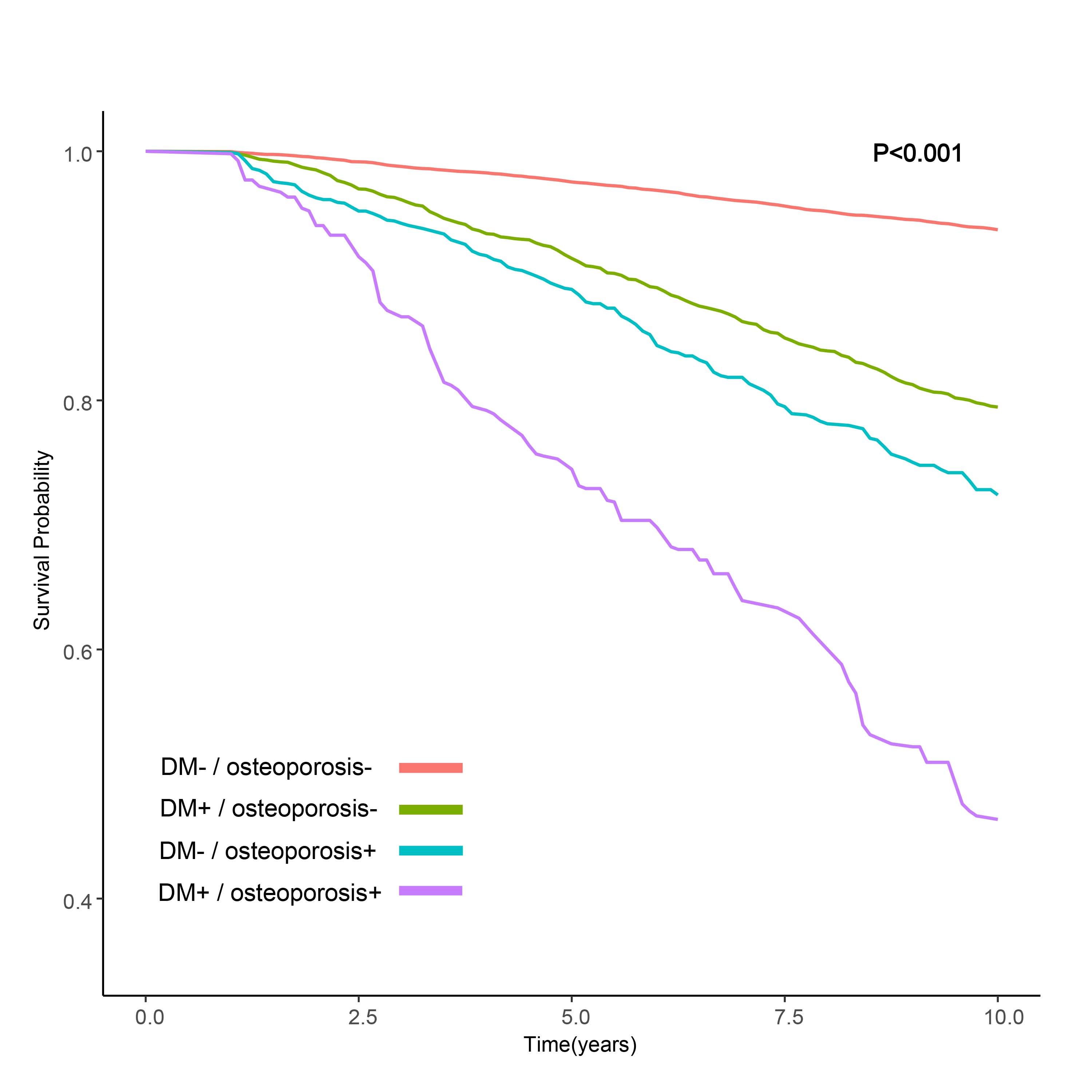

Supplement: Supplementary Figure 1 — Kaplan-Meier survival estimates for all-cause mortality (sensitivity analysis, weighted). [file Image_1.jpeg]
