# Supplementary material for: The synergistic effect of diabetes mellitus and osteoporosis on the all-cause mortality: a cohort study of an American population
Source: Front Endocrinol (Lausanne). 2024 Jan 24;14:1308574. doi: 10.3389/fendo.2023.1308574 (PMC10849060; doi:10.3389/fendo.2023.1308574)
Supplement: Supplementary file 2 [file Table_1.docx]

**Table S1. The association between diabetes and osteoporosis and all-cause death (weighted)**

| Group | Model 1 | | | Model 2 | | | | | Model 3 | | | | |  |
| --- | --- | --- | --- | --- | --- | --- | --- | --- | --- | --- | --- | --- | --- | --- |
|  | OR (95%CI) | *P-value* | | | OR (95%CI) | | *P-value* | | | OR (95%CI) | | *P-value* | |  |
| DM- / osteoporosis- | Ref | |  | | | Ref | |  | | | Ref | |  | |
| DM+ / osteoporosis- | 3.51(3.13-3.95) | | <0.001 | | | 1.60(1.43-1.78) | | <0.001 | | | 1.35(1.14,1.61) | | <0.001 | |
| DM- / osteoporosis+ | 4.71(3.64-6.10) | | <0.001 | | | 1.80(1.38-2.33) | | <0.001 | | | 1.59(1.08,2.33) | | 0.020 | |
| DM+ / osteoporosis+ | 10.88(7.71-15.37) | | <0.001 | | | 3.73(2.70-5.16) | | <0.001 | | | 2.33(1.61,3.38) | | <0.001 | |
| **Additive interaction** |  | |  | | |  | |  | | |  | |  | |
| RERI (95%CI) for DM and osteoporosis: 0.86(0.43-1.29) | | | | | | | | | | | | | | |
| AP (95%CI) for DM and osteoporosis: 0.36(0.27-0.45) | | | | | | | | | | | | | | |

Model 1: Not adjusted.

Model 2: Adjusted by age, gender, race/ethnicity.

Model 3: Adjusted by age, gender, race/ethnicity, education, smoke, drink, obese, Exercise metabolic equivalent, HbA1C, Phosphorus, Hyperlipidemia, CKD, Hypertension, CVD.
